# The salt-taste threshold in untreated hypertensive patients

**DOI:** 10.1186/s40885-017-0079-8

**Published:** 2017-11-15

**Authors:** Chang-Yeon Kim, Mi-Kyung Ye, Young Soo Lee

**Affiliations:** 1 Division of Cardiology, Daegu Catholic University Medical Center, 3056-6, Daemyung-4-dong, Nam-gu, Daegu, Korea; 2 Departments of Otorhinolaryngology-Head and Neck Surgery, Daegu Catholic University Medical Center, Daegu, Korea

**Keywords:** Taste, Sodium, Hypertension

## Abstract

**Background:**

The salt-taste threshold can influence the salt appetite, and is thought to be another marker of sodium intake. Many studies have mentioned the relationship between the sodium intake and blood pressure (BP). The aim of this study was to evaluate the relationship between the salt-taste threshold and urinary sodium excretion in normotensive and hypertensive groups.

**Methods:**

We analyzed 199 patients (mean age 52 years, male 47.3%) who underwent 24-h ambulatory BP monitoring (ABPM). Hypertension was diagnosed as an average daytime systolic BP of ≥135 mmHg or diastolic BP of ≥85 mmHg by the ABPM. We assessed the salt-taste threshold using graded saline solutions. The salt-taste threshold, 24-h urinary sodium and potassium excretion, and echocardiographic data were compared between the control and hypertensive groups.

**Results:**

The detection and recognition threshold of the salt taste did not significantly differ between the control and hypertensive groups. The 24-h urinary sodium excretion of hypertensive patients was significantly higher than that of the control group (140.9 ± 59.8 vs. 117.9 ± 57.2 mEq/day, respectively, *p* = 0.011). Also, the urinary sodium-potassium ratio was significantly higher in the hypertensive patients. There was no correlation between the salt-taste threshold and 24-h urinary sodium excretion.

**Conclusions:**

The salt-taste threshold might not be related to the BP status as well as the 24-h urinary sodium excretion.

## Background

It is indisputable that an elevated blood pressure (BP) is one of the most important risk factors for cardiovascular disease, heart failure, strokes, chronic kidney disease, peripheral arterial disease, and atrial fibrillation [1]. Many factors including the genetics, life style, medications, obesity, and dietary factors contribute to the development of hypertension in a complicated way.

Epidemiological studies have revealed that excessive salt intake plays a major role in elevating the BP in the global population, leading to increased cardio- and cerebrovascular morbidity and mortality [2–4]. Also, many studies have demonstrated that salt itself has an impact on the aortic stiffness and coronary heart disease in a BP-independent manner [3, 5]. The global mean sodium intake in 2010 was about 4 g/d (salt ≈ 10 g/d) in adults, and is especially higher in Asian regions [4]. In that year, the Korean population aged 20 and over had a high estimated sodium intake of 5.21 (4.98–5.48) g/d. WHO (World Health Organization) recommends a sodium reduction of <2 g/d in adults [6] and also the current hypertension guidelines recommend reducing the sodium intake as a way of a lifestyle modification [7].

If a high salt intake of the population has such a big impact on the global health, it is of importance to figure out what factors contribute to that high salt intake, which obviously is much more than needed. We were interested in the salt-taste sensitivity threshold, which meant how sensitively an individual perceives a salt-taste. When an individual has a high threshold for a salt-taste, it might be possible to expect that he or she would intake more salt than one who had a low threshold. There is little data about the relationship among the salt-taste sensitivity, amount of salt intake, and prevalence or incidence of hypertension. Even the limited data available has had inconsistent results [8–10]. Therefore, we tried to figure out if there were any differences in the salt-taste sensitivity threshold and amount of salt intake, estimated by the urinary sodium excretion, between normotensive and hypertensive groups.

## Methods

### Study population

We assessed 289 patients, aged 18 years or older, who visited our clinic for high BP from March 2013 to August 2016. The patients with diabetes mellitus (*n* = 8), pregnancy (*n* = 21), an impaired renal function (serum creatinine >1.5 mg/dL), and secondary hypertension such as hyperaldosteronism, Cushing’s syndrome, renal artery stenosis and thyroid dysfunction were excluded. Also, the patients whose ABPM (*n* = 12) or taste threshold (*n* = 47) data were missing, and who did not stop their medication (*n* = 2) were excluded. Finally, a total of 199 patients were included in the analysis. We checked thyroid hormones and plasma renin activity, serum aldosterone levels to exclude secondary hypertension. They were divided into control (normotension) and hypertension groups according to their ABPM results. They did not take any hypertensive medications. If they started to take hypertensive medications recently, they were included after cessation of the drug at least for 1 week. We compared the serum level of the sodium (Na), potassium (K), renin activity, aldosterone, blood urea nitrogen (BUN), and creatinine (Cr) between the two groups. This study was approved by the institutional review board of Daegu Catholic Medical Center.

### The definition of hypertension

Hypertension was diagnosed by 24-h ABPM (TM-2430, A&D company, Tokyo, Japan), that is, an average daytime systolic BP of ≥135 mmHg or diastolic BP of ≥85 mmHg. The patients were checked for nocturnal BP dipping patterns and were divided into 4 groups: reverse-dipper (rise in the nocturnal BP), non-dipper (<10%), dipper (10~20%), and extreme-dipper (>20%). The morning BP surge was calculated as the morning BP (after awakening, average of 3 consecutive BP readings) minus the nocturnal BP (average 3 consecutive BP readings centered on the lowest nocturnal BP).

### The measurement of the salt-taste threshold

The salt-taste threshold was assessed, using graded solutions of saline as shown in Table 1. We employed a whole-mouth gustatory test procedure to test the solutions. Each level of solution was applied to the tongue evenly (1 ml of the solution with a 5 ml syringe). Then, the subject held the solution for a few seconds and swallowed it. The subject was then asked about the taste of the solutions. Starting from the lowest concentration, the detection threshold was defined as the point at which the subject perceived a different taste from that of the distilled water. The recognition threshold was defined as the lowest concentration at which the subject recognized the solution as being salty. We went back to a lower concentration and repeated the test again if the threshold could not be determined. The subjects rinsed their mouth with water whenever the subject tested a different concentration of the solution. We performed the same test once again another day in patients with an unusual test result (*n* = 14).

**Table 1:** The concentration of the salt-taste solution

Concentration levels of the taste solution	Sodium chloride (g/mL)
1	0.00488
2	0.00977
3	0.01953
4	0.03906
5	0.07813
6	0.15625
7	0.31250
8	0.62500
9	1.25000
10	2.50000
11	5.00000
12	10.00000
13	20.00000

### The 24-h urinary sodium and potassium excretion

We collected 24-h urine samples and measured the sodium excretion in it to estimate the amount of sodium intake. The patients were instructed to collect self-urine samples for 24 h after discarding the first morning urine. The urine samples were kept in a refrigerator at home and brought to the laboratory.

### Transthoracic echocardiography

The left ventricular (LV) chamber diameters were acquired in the parasternal long-axis view and left atrial (LA) chamber diameters in the parasternal short-axis view. The LV mass was calculated using a 2D–guided M-mode method where the following parameters were included: interventricular septum, posterior wall thickness, and LV internal diameter at end-diastole. The LA volume was calculated using a disk summation technique. The ejection fraction (EF) was measured using the biplane method of disks and M-mode of the LV in the parasternal long-axis view. For the analysis, we chose one method, the M-mode method for convenience. The LV mass index (LVMI) was calculated by dividing the LV mass by the body surface area (BSA) and the LA volume index (LAVI) by dividing the LA volume by the BSA.

### Statistical analysis

The differences in the baseline characteristics of the patients in each group were compared with the use of a Pearson’s chi-square test for categorical variables. A Student’s *t*-test was used for a comparison of the continuous variables. To assess the relationship among the continuous variables, correlation and regression analyses were used. Spearman correlation method was used to assess the relationship between ordinal variable and continuous variable. The one-way analysis of variance (ANOVA) test was used to compare the data among the taste threshold groups and BP groups. The Bonferroni’s method was used as a post hoc analysis. Multiple regression analysis was used to assess the relationship between the LVMI and the salt taste threshold. The values are expressed as the mean ± standard deviation. *P* values of <0.05 were considered significant. All analyses were conducted using IBM SPSS statistics software (version 19.0, Chicago, IL, USA).

## Results

### Patient characteristics

A total of 199 patients were included in our study and their characteristics are shown in Table 2. The proportion of males was significantly higher in the hypertensive group than control group. The age, BMI, and BSA were similar between the two groups. The serum sodium and potassium levels were similar between the two groups. Also, the other laboratory test results, including the BUN, Cr, plasma renin activity and aldosterone levels, were similar between the two groups.

**Table 2:** The patient characteristics

Variables	Control	HTN	*p*
Patient number	66	133	
Male, n (%)	22 (33.3%)	71 (53.4%)	0.010
Age, years	53.6 ± 12.5	51.7 ± 10.9	0.273
Height, cm	162.4 ± 9.5	165.1 ± 8.9	0.054
Weight, kg	66.0 ± 12.9	68.8 ± 11.5	0.115
BMI, kg/m^2^	24.8 ± 3.2	25.2 ± 3.2	0.492
BSA, m^2^	1.72 ± 0.21	1.77 ± 0.18	0.073
Serum sodium, mEq/L	140.0 ± 2.4	139.7 ± 2.6	0.390
Serum potassium, mEq/L	4.47 ± 0.37	4.36 ± 0.36	0.057
Blood urea nitrogen, mg/dL	13.2 ± 3.8	13.1 ± 3.5	0.858
Serum creatinine mg/dL	0.74 ± 0.15	0.78 ± 0.16	0.150
Urinary sodium, mEq/d	117.9 ± 57.2	140.9 ± 59.8	0.011
Urinary potassium, mEq/d	52.2 ± 20.8	50.4 ± 24.2	0.638
Urinary sodium/potassium	2.46 ± 1.07	3.03 ± 1.18	0.003
Plasma renin activity, ng/ml/h	1.85 ± 1.53	1.83 ± 1.41	0.936
Serum aldosterone, pg/ml	89.6 ± 68.9	79.4 ± 55.1	0.278

*HTN* hypertension, *BMI* body mass index, *BSA* body surface area

### Transthoracic echocardiography

The summaries of the echocardiographic data are shown in Table 3. The LV systolic function was normal or nearly normal in the entire study population. The LVMI correlated with the daytime systolic/diastolic BP (*p* = 0.001, *r* = 0.244 and *p* = 0.011, *r* = 0.183, respectively). The average LVMI in the hypertensive group was significantly higher than that in the control group. When the LVMI was analyzed separately by the gender, it did not significantly differ between the control and hypertension groups in males (128.8 ± 20.4 vs. 133.5 ± 25.6, respectively, *p* = 0.435) as well as females (114.6 ± 28.2 vs. 121.0 ± 23.5, respectively, *p* = 0.21). There was no correlation between the LVMI and the recognition threshold of salt taste, adjusted by systolic BP (partial correlation coefficient = 0.117, *p* = 0.103). When the subjects were divided into three groups according to the recognition threshold of salt, the LVMI differed significantly between groups (Table 3). In post hoc analysis, there was significant difference in the LVMI between the subjects with threshold of 7 and subjects with threshold 8 or more (*p* = 0.030). But there was marginal significance between the subjects with threshold of 7 and the subjects with 6 or less (*p* = 0.071). The other echocardiographic parameters, including the LV dimension, LA dimension, and LAVI did not differ between the control and hypertension groups.

**Table 3:** The echocardiographic measurements

Variables	Control	HTN	*p*	Recognition threshold of salt taste			*p*
				<7 (*n* = 95)	7 (*n* = 71)	>7 (*n* = 33)	
LVEF, %	64.5 ± 6.0	63.6 ± 5.7	0.296	63.9 ± 6.1	64.4 ± 5.3	63.0 ± 6.0	0.542
LVEDD, mm	49.4 ± 4.7	50.5 ± 5.1	0.128	49.9 ± 5.1	50.1 ± 4.7	50.8 ± 5.2	0.681
LVESD, mm	31.8 ± 4.2	32.8 ± 4.2	0.110	32.3 ± 4.4	32.3 ± 3.9	33.1 ± 4.2	0.577
LAD, mm	36.7 ± 4.6	37.9 ± 4.6	0.073	37.0 ± 4.3	37.7 ± 4.9	38.3 ± 5.0	0.353
LVMI, g/m^2^	119.4 ± 26.5	127.6 ± 25.3	0.036	123.6 ± 24.8	121.4 ± 21.9	135.4 ± 34.1	0.030
LAVI, ml/m^2^	27.0 ± 7.7	28.1 ± 8.1	0.382	27.4 ± 7.6	28.1 ± 7.9	27.9 ± 8.9	0.843

*LVEF* left ventricular ejection fraction, *LVEDD/LVESD* left ventricular end diastolic/systolic dimension, *LAD* left atrial dimension, *LVMI* left ventricular mass index, *LAVI* left atrial volume index

### The 24-h ambulatory blood pressure monitoring

An average daytime BP in the hypertension group was not so high (Table 4). Only 31 patients had an average systolic daytime BP of ≥155 mmHg or higher. The BP difference between the two groups was about 20 mmHg for the systolic pressure. The degree of nocturnal dipping was a little higher in the hypertension group than the control group, but there was no statistical significance. The non-dipper group contributed to 50.0% of the control group and 46.6% of the hypertension group. Nocturnal dipping was not related to the status of hypertension (*p* = 0.459). Further, the degree of morning BP surge was similar between the two groups. When we divided the subjects into three groups according to the recognition threshold of salt taste, the ABPM data did not differ among groups (Table 4).

**Table 4:** The 24-h ambulatory blood pressure monitoring data

Variables	Control	HTN	*p*	Recognition threshold of salt taste			*p*
				<7 (*n* = 95)	7 (*n* = 71)	>7 (*n* = 33)	
Mean SBP, mmHg	125.0 ± 6.7	145.1 ± 12.1	< 0.001	138.8 ± 13.9	136.5 ± 13.6	141.7 ± 16.1	0.205
Mean DBP, mmHg	78.8 ± 5.7	92.5 ± 9.5	< 0.001	88.0 ± 10.8	86.7 ± 9.7	90.7 ± 11.8	0.194
SBP at day, mmHg	127.2 ± 6.5	148.1 ± 11.2	< 0.001	141.7 ± 13.7	139.4 ± 13.5	143.4 ± 15.6	0.339
DBP at day, mmHg	79.8 ± 4.4	94.8 ± 9.1	< 0.001	90.0 ± 10.9	88.6 ± 9.6	91.7 ± 11.6	0.367
SBP at night, mmHg	117.9 ± 10.3	134.8 ± 16.5	0.001	129.7 ± 17.7	127.1 ± 15.9	132.2 ± 15.5	0.331
DBP at night, mmHg	73.1 ± 7.4	85.1 ± 12.0	< 0.001	81.7 ± 12.7	79.1 ± 11.4	83.7 ± 11.1	0.154
MBPS, mmHg	18.7 ± 15.6	19.1 ± 13.6	0.485	19.5 ± 12.6	19.0 ± 15.6	17.2 ± 16.1	0.721
Nocturnal dipping			0.199				0.527
Reverse-& non-dipper, n (%)	43 (65.2%)	74 (55.6%)		56 (58.9%)	39 (54.9%)	22 (66.7%)	
Dipper & extreme-dipper, n (%)	23 (34.8%)	59 (44.4%)		39 (41.1%)	32 (45.1%)	11 (33.3%)	

*HTN* hypertension, *S/DBP* systolic/diastolic blood pressure, *MBPS* morning BP surge

### The 24-h urinary sodium and potassium excretion

**Fig. 1:**
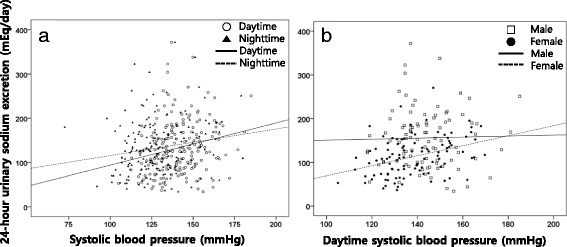
The relationship between the systolic blood pressure (BP) and 24-h urinary sodium excretion **a** and comparison by gender (**b**)

The 24-h urinary sodium excretion and Na/K ratio were significantly higher in the hypertensive group compared to the normotensive group, but the 24-h urinary potassium excretion did not differ between the two groups (Table 2). The 24-h urinary sodium excretion was correlated to the average systolic BP during the daytime and nighttime (Fig. 1a, p=0.002, *p* = 0.018, respectively). The 24-h urinary sodium excretion was higher in the male than female group (155.9±67.3 vs. 113.5±44.0 mEq/d, respectively, *p* < 0.001). When the 24-h urinary sodium excretion was analyzed separately by the gender, it was correlated to the BP only in females and not in males (Fig. 1b, p<0.001, *p* = 0.825, respectively). There was no correlation between the degree of nocturnal dipping and the urinary sodium excretion (Spearman’s rho=0.076, *p*=0.294). After adjusting the systolic BP, the LVMI was correlated to the 24-h urinary sodium excretion (*p*=0.032, *r*=0.156).

### The salt-taste threshold

**Fig. 2:**
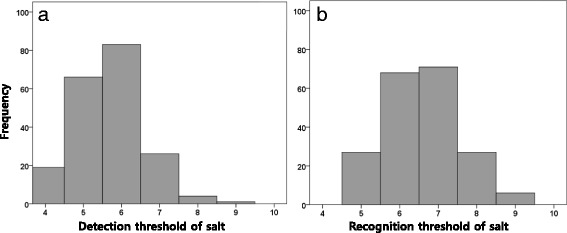
Histogram of the salt taste threshold

**Fig. 3:**
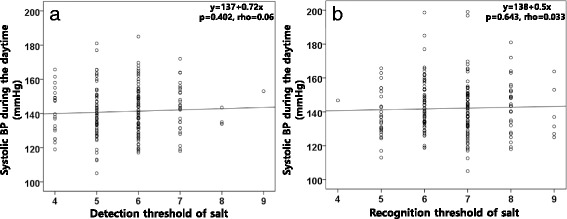
The relationship between the detection (**a**) and recognition (**b**) threshold of salt and daytime systolic blood pressure (BP)

**Fig. 4:**
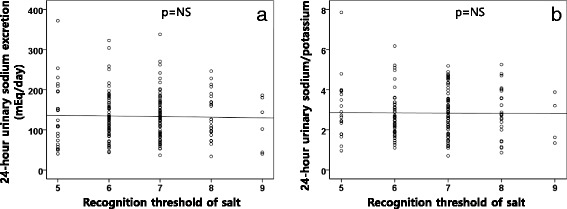
The relationship between the 24-h urinary sodium excretion (**a**) and 24-h urinary sodium/potassium ratio (**b**) and recognition threshold of salt

The salt-taste thresholds did not significantly differ between the control and hypertension groups (Table 5), and also between genders. More than two-thirds of the study population (69.8%) corresponded to a recognition threshold of 6 and 7 (Fig. 2). Also, there were no correlations between the detection and recognition thresholds and the daytime systolic BP (Fig. 3). The 24-h urinary sodium excretion did not show any correlation to the detection and recognition threshold of salt (Fig. 4). When we divided the patients into 3 groups according to the degree of the BP status, the recognition threshold of salt did not differ (*p* = 0.918 in one-way ANOVA). Also, the recognition threshold of salt did not correlate with the early morning BP surge (Spearman’s rho = −0.080, *p* = 0.26) and was not associated with dipping patterns; extreme dipper, dipper, non-dipper, and reverse dipper (Spearman’s rho = −0.007, *p* = 0.925).

**Table 5:** The salt-taste threshold

Variables	Control	HTN	*p*
Detection threshold	5.65 ± 0.87	5.67 ± 0.96	0.900
Recognition threshold	6.56 ± 1.03	6.59 ± 0.97	0.823

*HTN* hypertension

## Discussion

In our study, both the detection and recognition threshold of salt had no relationship to the 24-h urinary sodium excretion, which is an estimate of the sodium intake. The systolic and diastolic BPs positively correlated with the 24-h urinary sodium excretion, and that relationship persisted in females, but not in males. Further, the salt-taste thresholds in the hypertensive group did not differ from those in the control group.

### Salt taste-threshold and hypertension

There are some controversies about the relationship between the salt-taste threshold and BP status. Some studies have reported that hypertensive patients have an elevated recognition threshold for salt [11–14], but other studies have not noted any difference in the recognition threshold between normotensive and hypertensive persons [15, 16]. Because most of those studies were conducted in a small population, Fischer et al. assessed the relationship in a quite large study population with over 2000 people [8]. They reported that there was no significant association between hypertension and the salt taste intensity, and the salt taste intensity of males was less strong than that of females. Similarly, in our study, the detection and recognition thresholds for salt did not differ between the control and hypertension groups. It is probably because people with a recognition threshold level of 6 and 7 accounted for nearly 70% of the total study population, which meant that it may be hard to discern the differences between the groups. Although there are still controversies about the association between the salt-taste sensitivity and blood pressure, to date, there is little evidence to say that hypertensive patients have a higher salt-taste threshold than normotensive people.

### Salt recognition threshold and urinary sodium excretion

Other studies concerning the relationship between the amount of salt intake and salt-taste sensitivity showed that there was no significant association, but their study populations were healthy volunteers [17–19]. In agreement with their studies, no correlation was observed between the 24-h urinary sodium excretion and recognition threshold. One explanation may be that the salt-taste sensitivity itself did not seem to affect the individual’s habitual salt intake. This may be because the majority of the source of an individual’s sodium intake is determined by food processing steps not by discretionary salt use [20]. Various dietary sodium sources such as the discretionary use of cooking and table salt, inherent salt in food, added salt in processing, etc. comprise an individual’s total sodium intake. If more than two thirds of the total intake of salt is determined regardless of an individual’s preference for salt, it is a natural result that the salt-taste sensitivity does not affect a person’s total sodium intake. Moreover, there is no consistent evidence about the association between the salt preference and actual salt intake [16, 18, 21, 22]. In this regard, only an individual’s effort to reduce the sodium intake has a critical limitation.

### Urinary sodium excretion and the blood pressure

There are lots of epidemiological, migratory and experimental studies that have shown a positive relation between the amount of salt intake and the BP [23, 24]. Similar to the INTERSALT (International Cooperative Study on Salt, Other Factors, and Blood Pressure) study, the 24-h urinary sodium excretion was significantly higher in the hypertensive group than control group in our study. Migrants to high salt intake areas had a higher BP and greater increase in the BP along with an increasing age than did their relatives left behind [25, 26]. Moreover, there are many studies showing that a reduction in the salt intake in a population led to a decrease in the BP in the community [27, 28].

In most epidemiological studies about salt intake, males tend to have a higher urinary sodium excretion than females [29]. The exact reasons for the difference are not known but in part, there is a higher amount of food intake and higher body weight in males [30]. Our data are in agreement with these findings. The daytime systolic BP had a positive correlation to the urinary sodium excretion in females, but not in males. One of the reasons may be that a female’s blood pressure is more sensitive to salt loading than a male’s. Kojima et al. observed a BP reduction only in females when the dietary sodium intake was dramatically decreased [31]. Also, He et al. reported greater BP responses in females compared to males in dietary sodium intervention [32]. Females tend to have a lower body weight than males and especially during the postmenopausal period, the change in the composition of the sexual hormones, like estrogen, and testosterone, may also affect the salt sensitivity to the BP response [33].

### Limitations

This study had several limitations to consider. First, the taste thresholds were distributed relatively in a small range so it was hard to tell whether the differences between the groups existed or not. Therefore, a larger study is needed to evaluate the accurate relationship between the taste threshold and BP. Second, the weakness of the 24-h urine collection method places a heavy burden on the patients. It is not easy to collect the total 24-h urine correctly for socially active persons. Unfortunately, we could not check the 24-h urine creatinine to see if the urine samples were adequately collected. Further, an individual’s daily sodium intake varies day by day. Ideally, several 24-h urine collections are needed to estimate an individual’s accurate sodium intake. Because of the difficulty in collecting 24-h urine samples, many studies have inferred the 24-h urinary sodium excretion using Tanaka’s method or Kawasaki’s method from spot urine sample, or using a food frequency questionnaire [34, 35]. Thus far, 24-h urine collections have been known to be the gold standard for evaluating the 24-h urinary sodium excretion as compared to the various method using spot urine samples. Third, we obtained urine samples after explaining the impact of the salt intake on hypertension. That could influence the patient’s salt intake habits. In Korea in 2014, the average sodium intake per capita was 3.9 g/d (salt equivalent 9.72 g/d) [36]. However, in our study, the average 24-h urinary sodium excretion was 3.1 g (133.2 mEq)/d, that is a much lower sodium intake amount. That was inevitable in order to conduct the study and to receive a written consent from patients. Finally, this was a single center study and had small study population. Therefore, multicenter and large scaled studies are required to precisely evaluate the relationship among the salt-taste threshold, 24-h urinary sodium excretion, and BP.

## Conclusions

The salt-taste threshold might not be related to the BP status as well as the 24-h urinary sodium excretion.
